# Semantic relational machine learning model for sentiment analysis using cascade feature selection and heterogeneous classifier ensemble

**DOI:** 10.7717/peerj-cs.1100

**Published:** 2022-09-20

**Authors:** Anuradha Yenkikar, C. Narendra Babu, D. Jude Hemanth

**Affiliations:** 1 Department of Computer Science and Engineering, M. S. Ramaiah University of Applied Sciences, Bengaluru, Karnataka, India; 2 Department of Electronics and Communications Engineering, Karunya University, Coimbatore, Tamil Nadu, India

**Keywords:** Sentiment analysis, Deep learning, Natural language processing, Ensemble model

## Abstract

The exponential rise in social media *via* microblogging sites like Twitter has sparked curiosity in sentiment analysis that exploits user feedback towards a targeted product or service. Considering its significance in business intelligence and decision-making, numerous efforts have been made in this area. However, lack of dictionaries, unannotated data, large-scale unstructured data, and low accuracies have plagued these approaches. Also, sentiment classification through classifier ensemble has been underexplored in literature. In this article, we propose a Semantic Relational Machine Learning (SRML) model that automatically classifies the sentiment of tweets by using classifier ensemble and optimal features. The model employs the Cascaded Feature Selection (CFS) strategy, a novel statistical assessment approach based on Wilcoxon rank sum test, univariate logistic regression assisted significant predictor test and cross-correlation test. It further uses the efficacy of word2vec-based continuous bag-of-words and n-gram feature extraction in conjunction with SentiWordNet for finding optimal features for classification. We experiment on six public Twitter sentiment datasets, the STS-Gold dataset, the Obama-McCain Debate (OMD) dataset, the healthcare reform (HCR) dataset and the SemEval2017 Task 4A, 4B and 4C on a heterogeneous classifier ensemble comprising fourteen individual classifiers from different paradigms. Results from the experimental study indicate that CFS supports in attaining a higher classification accuracy with up to 50% lesser features compared to count vectorizer approach. In Intra-model performance assessment, the Artificial Neural Network-Gradient Descent (ANN-GD) classifier performs comparatively better than other individual classifiers, but the Best Trained Ensemble (BTE) strategy outperforms on all metrics. In inter-model performance assessment with existing state-of-the-art systems, the proposed model achieved higher accuracy and outperforms more accomplished models employing quantum-inspired sentiment representation (QSR), transformer-based methods like BERT, BERTweet, RoBERTa and ensemble techniques. The research thus provides critical insights into implementing similar strategy into building more generic and robust expert system for sentiment analysis that can be leveraged across industries.

## Introduction

In the last few years, soft computing and internet technologies have emerged as an inevitable tool for business. Strategic implementation of these technologies has broadened the horizon for businesses to exploit it in making optimal business decisions, be it marketing, new product development, quality optimization and recommendations (Pang & Lee, 2008; Davies & Ghahramani, 2011; Prabowo & Thelwall, 2009; Collomb et al., 2014). One of the important technologies that have drawn significant attention is Natural Language Processing (NLP) that automatically exploits customer or user’s perception towards certain product, service, event etc. to make optimal decisions. NLP has given rise to a new paradigm called sentiment analysis which is also known as opinion mining. It is the management of sentiments, views, and subjective material (Yeole, Chavan & Nikose, 2015). Sentiment analysis deals with the process of analyzing several tweets and reviews to provide comprehensive information on public opinion. It is a tried-and-tested tool for predicting a wide range of key events, including boxing matches, movie box office receipts and general elections (Heredia et al., 2016). Public reviews are used to assess a specific thing, such as a person, product or a destination, and it can be found on a variety of websites. Opinions can be divided into three categories, negative, positive, or neutral. The goal of sentiment analysis is to find out how people feel and aims to determine the user’s expressive direction automatically (Luo, Li & Cao, 2016). Sentiment analysis is becoming more popular as the need for analyzing and structuring hidden information from social media in the form of unstructured data grows (Haenlein & Kaplan, 2010). Though its significance towards making suitable bureaucratic or government decisions has not yet been explored, however a strategically deployed sentiment analysis model can help major stakeholders make optimal target-centric decisions (Iqbal et al., 2019).

Realizing its potential, numerous efforts have been made on analyzing sentiments by exploiting lexicon based (Taboada et al., 2011) and rule based (Collomb et al., 2014) approaches. Off-late major efforts in machine learning (ML) have played a decisive role in performing training and learning over the association between sentiment words and other aspects (Pang & Lee, 2008; Davies & Ghahramani, 2011; Prabowo & Thelwall, 2009; Collomb et al., 2014; Iqbal et al., 2019). In addition, machine learning becomes more apt for sentiment analysis due to its ability to perform global classification rather than individual aspects of the reviewed service or product (Iqbal et al., 2019). Existing ML techniques have applied labeled dataset for classifier training that eventually determines the sentiment (Taboada et al., 2011). Lexicon-based models involve estimating sentiment polarity on feedbacks or reviews by means of semantic orientation of words in a complete text-sentence. On the other hand, rule-based methods explore the opinion words in text and use ML concept to classify into categories (Taboada et al., 2011; Ding, Liu & Yu, 2008). However, complex user-dependent text data and unannotated context make such approaches limited. In addition, the use of data specific rules, dictionary polarity, negation words, idioms etc. makes it even more complex (Iqbal et al., 2019). In the last few years, semantic feature-based text analysis approaches have gained widespread attention across academia and industry. Many classical semantic analysis paradigms use bag-of-words (BoW) feature for classification. In addition, different approaches employing BoW have exploited *n*-gram, boolean, co-occurrence and TF-IDF to perform sentiment analysis. However, training and feature selection has always been a major hurdle for these approaches. As an alternate, word2vec (Mikolov et al., 2013) features have exhibited better performance however, the need for a suitable ML classifier has always been a necessity. Though word2vec with *n*-gram models have been applied for feature extraction however, they are unable to solve data sparsity problem. Rare word embeddings also make conventional word2vec limited for sentiment analysis tasks. Reducing features and training them with suitable learning methods have always been a challenge for the research community. To perform sentiment analysis, authors have tried to use different ML algorithms such as Neural Networks, SVM, NB, DT etc. There have also been some efforts to implement Ensemble strategy. Oscar et al. (2017) proposed several models where classic hand-crafted features are combined with automatically extracted embedding features, as well as the ensemble of analyzers that learn from these varied features with good results. da Silva, Hruschka & Hruschka (2014) used Multinomial Naive Bayes, SVM, Random Forest, and Logistic Regression classifiers in an ensemble to improve classification accuracy. Lochter et al. (2016) presented an ensemble method to determine an optimal way to combine cutting-edge text processing techniques, such as language normalization and semantic indexing, with traditional classification methods to detect opinions in brief text messages automatically. For sentiment analysis and opinion mining, Kumar et al. (2021) proposed a Drift Detection-based Adaptive Ensemble classifier, which benefits from false-positive drift detection signals while minimizing their negative influence. Whenever there is a drift in the ensemble, the proposed technique builds and adds a new classifier. Cambria (2016) looked at effective computing and sentiment analysis using the ever-increasing social data available online. However, in practice, majority of these approaches have either been applied for small scale data or for annotated details. On the contrary, the exponential rise in unannotated and unstructured data demands more effective solutions with better data processing, feature selection and classification (Iqbal et al., 2019). Major conventional ML methods undergo local minima and convergence thus limiting their efficacy for large scale heterogeneous data processing for sentiment analysis tasks. Considering the above limitations, this study focusses on developing a highly robust and enhanced sentiment analysis system that exploits the efficacy of semantic features, advanced feature selection and ensemble classification. Unlike major conventional approaches where authors have either focused on feature extraction or classification using machine learning and deep learning techniques, in Semantic Relational Machine Learning (SRML) model, emphasis is on augmenting each step of NLP to accomplish a robust sentiment analysis paradigm. However, in large datasets where there can be significantly large number of words (often called non-sentiment or rare-words) having no significance towards sentiment prediction, proposed is a novel multi-phase cascaded feature selection (CFS) model that uses Wilcoxon rank sum, ULR-assisted significant predictor test, and cross-correlation tests. The proposed CFS model ensures that the proposed system retains only significant or optimal features for sentiment classification. Word2vec with continuous bag-of-words (CBOW) and n-gram is used to extract semantic features. Unlike classical word2vec, our proposed SRML model incorporates CBOW with 1- and 2-gram methods to obtain features, which has later been processed in conjunction with SentiNetWord to enable weighing of each sentiment. A total of 14 base classifiers from different paradigms in standalone mode and in an ensemble model are implemented using four public datasets with Twitter review data. Overall, the key contributions from this article are:

- A novel cascade feature selection approach that can support higher classification accuracy with only significant or optimal features feeding the SRML mode
- A Semantic Relational Machine Learning (SRML) model using classifier ensemble strategy to evaluate sentiment classification by comparing performance with state-of-the-art systems across six datasets.

The manuscript is divided as follows: next section discusses related literature pertaining to sentiment analysis and latest research. Next, we explain the method and materials used in the development of the system. We summarize the results and discuss the findings. This is followed by conclusions and future research directions followed by references at the end of the manuscript.

## Related literature

There are different approaches proposed towards sentiment analysis, which are broadly categorized into rule-based, aspect-based and ML-based approaches. However, exploiting rule-based approaches in conjunction with machine learning has resulted in more efficiency. This section primarily discusses some of the key literature pertaining to sentiment analysis. Carvalho & Plastino (2016) provide literature study of feature representation in Twitter sentiment analysis. Categorizing features that have comparable characteristics, the authors used feature selection algorithms to find relevant subsets of features in each dataset. Lexicon based approaches have been applied as an unsupervised method to perform sentiment analysis (Hu et al., 2013). They applied both synonym set and antonym set in WordNet to find semantic orientation. This approach was found suitable for identifying the words pertaining to certain specific sentiment class only. However, its computational overhead over large feedback or review data can’t be ignored. Also, it is highly dependent on language and allied defined lexicons, which isn’t effective to understand semantic features or to offer better sentiment accuracy. Peng & Shih (2010) used part-of-speech method (POS) to obtain sentiment phrases of the user’s review wherein they applied unknown phrases as input to assess sentiment. In addition, it used top-*n* appropriate phrases and amalgamated both the unknown phrases as well as the known phrases to perform lexicon-based sentiment analysis. However, its suitability over generic working environment seems limited. Kamps et al. (2004) used WordNet synonym chart to estimate semantic distance for word sentiment orientation estimation. Ding, Liu & Yu (2008) too used lexicon-based approach by comparing opinion words and linguistic rules which enable identification of the semantic orientations pertaining to product features. As regards rule-based model, Khan, Baharudin & Khairullah (2011) developed SentiWordNet that exploited polarity and score matrix of a phrase to predict sentiment. Though authors recommend their model as better alternative to machine learning methods, however accuracy of 76.8% and 86.6% at the feedback and sentence level respectively raises a question mark about its generalization. It motivates authors to develop more efficient solutions and explore enhanced machine learning approaches. Lee, Chen & Huang (2010) tried on similar lines and first developed an emotional dataset using a series of linguistics rules which was later processed for emotion cause detection. As regards machine learning methods, authors applied SVM and Conditional Random Field (CRF) algorithm for sentiment classification. Alfaro et al. (2016) used the concept of opinion mining and sentiment analysis to find out the various trends in weblogs. Authors found that SVM can be a potential alternative to KNN classifier to perform sentiment analysis. Authors used SVM algorithm to mine product reviews for different services and marketing activities to assess consumer’s sentiment. Baccianella, Andrea & Fabrizio (2010) used NB, SVM, and random forest algorithms for sentiment analysis, considering the success of machine learning in sentiment analysis tasks. To determine the sentiment of diverse texts, the authors used POS tags, word *n*-grams, and tweet context information elements such as hashtags, retweets, emoticons, capital terms, and so on. By evaluating the performance of several classifiers in terms of accuracy, Govindarajan (2013) created a hybrid sentiment classification model. For sentiment analysis, a hybrid classifier was created utilizing NB and Genetic Algorithm. Carvalho, Prado & Plastino (2014) proposed a statistical method to classify tweets which uses genetic algorithm to determine the pattern words. This algorithm looks over a list of pattern words to find a subset of them that improves classification accuracy considerably. Annett & Kondrak (2008) applied different algorithms such as SVM, NB classifier, alternating decision trees and lexical based approaches to perform sentiment analysis and they found that SVM achieves better accuracy (75%) than NB or decision tree. Boiy et al. (2007) on the contrary obtained 90.25% accuracy for car and movie reviews dataset. Authors found that SVM outperforms NB algorithm. Similar result was affirmed by Cui, Mittal & Datar (2006) as well who found SVM better than NB especially for large scale data, though (Yang et al., 2010) found that NB approach is highly time consuming and is effective only for small scale datasets.

With a motive to exploit machine learning models, Chen et al. (2006) applied Decision Tree, SVM and NB algorithms to review the book *The da Vinci Code* on amazon.com, where they found that SVM achieves maximum accuracy of 84.59%. Ye, Zhang & Law (2009) used same classifiers for sentiment analysis of tourist reviews on Yahoo.com and found that SVM outperforms other approaches with an accuracy of 85.14%. When applying SVM as sentiment classifier for movie review collected by IMDB, Palitglou & Thelwall (2010) achieved highest accuracy of 96.60%. SVM with hybrid features through inverse bias algorithm was used by Li & Xu (2014) to perform stock market sentiment analysis. Chinnalagu & Durairaj (2021), Gui et al. (2016) used SVM to recognize emotion and captured the cause information using convolution kernels from syntactic trees. In their sentiment analysis model, Li & Meesad (2016) used the k-means method to cluster the data into positive and negative groups, achieving a 70% accuracy. Considering buyers sentiment as an intelligent business decision, Schuster & Paliwal (1997) suggested bidirectional recurrent neural network model for sentiment classification. Lilleberg, Zhu & Zhang (2015) applied word2vec for text (sentiment) classification, wherein they found that word2vec outperforms TF-IDF. This motivates us to exploit word2vec with CBOW concept for optimal semantic feature extraction and further classification. To exploit optimal efficacy of word2vec, Go, Bhayani & Huang (2009) recommended tokenization and non-word tokenization. Authors found that this approach can enable suitable feature extraction and retention for further classification. Cambria (2016) extracted people’s feelings from an online social database using human-computer interaction, information retrieval, and multi-modal signal processing technologies. Carvalho & Plastino (2021) provided a complete evaluation of features using multiple supervised learning algorithms on twenty-two Twitter datasets. They tested many meta-features and pre-trained word embeddings in recent publications. They also assess and study the impact of mixing those various types of features to determine which combination may give important information in Twitter sentiment polarity identification task.

For sentiment analysis and classification, classifier ensemble and deep learning approaches have also been used in recent years. Alsayat (2022) proposed a customized deep learning model with an advanced word embedding technique and created a long short-term memory (LSTM) network. They also proposed an ensemble model that combines baseline classifier with other state-of-the-art classifiers used for sentiment analysis. Aziz & Dimililer (2020, 2021) proposed a novel ensemble classifier approach, which utilizes a combination of multiple feature sets by combining multiple individual classifiers, which are weak learners, into an ensemble classifier. The feature sets used included Bag of Words, Term Frequency—Inverse Document Frequency, Part of Speech, N-gram, Opinion Lexicon, and Term Frequency. Their experiments confirmed that the proposed ensemble method outperforms all individual classifiers and significantly improves the overall sentiment classification performance on the most frequently used datasets in Sentiment Analysis. Similar results on ensemble classification were reported by Alaa et al. (2020), Kazmaier & van Vuuren (2021) and hence our choice in this research combined with a novel feature selection technique. The convolutional neural network (CNN) (Kalchbrenner, Grefenstette & Blunsom, 2014; Santos & Gatti, 2014) and recurrent neural network (RNN) have also been used to achieve improved accuracy. Kim (2014) built a phrase level sentiment classification model by combining a pre-trained vector model with CNN. Johnson & Zhang (2014) employed a one-hot encoding technique and CNN to extract features for classification. By combining data augmentation approach with the pre-trained language model, Lu et al. (2021) created a unified model termed PEA. It also includes an ensemble method that produced good results by combining the results of the fundamental RNN-based and BERT-based models. Mehta, Pandya & Kotecha (2021) developed and deployed a stock price prediction accuracy tool that considered public mood and used machine learning algorithms and Long Short-Term Memory to achieve the best accuracy of 92.45%. Chinnalagu & Durairaj (2021) suggested a high-performance yet cost-effective model that classified text and word embedding utilizing the fastText package from Facebook’s AI research (FAIR) Lab, as well as the standard Linear Support Vector Machine (LSVM). The accuracy of the fastText model achieved is 90.71%. Barreto et al. (2021) presented an evaluation of text representation models from the classical to the modern era is carried out. Challenges in linguistic styles for NLP and sentiment analysis are addressed. Trendy BERT architecture and 22 datasets from distinct domains and five classification algorithms are used. Chinatalapudi, Battineni & Amenta (2021) proposed a Deep Learning (DL) based analysis of Indian covid-19 tweets through the lock down period. Different emotions were analyzed using Bi-Directional Encoder Representation from Transformers (BERT) and results compared with traditional logistic regression (LR), support vector machines (SVM), and long short-term memory (LSTM). Accuracy of BERT model, LR, SVM, LSTM are 89%, 75%, 74.75% and 65% respectively. Jiang et al. (2022) suggested a hybrid model (BERT-BiLSTM-TextCNN) to obtain better accuracy on text-based psychological analysis of online comments. BiLSTM and TextCNN are used to capture local correlation, while BERT is used to produce word vectors in this model. Mohammad et al. (2021) suggested a bidirectional CNN-RNN deep model based on attention (ABCDM). Past and future perspective were considered by two layers of BiLSTM and GRU layers. For ABCDM bidirectional layer’s output, an attention model is used to emphasize various words simultaneously. To analyze text sentiments (Ko & Chang, 2021) used the NLP tool BERT and LSTM for evaluating time series data to anticipate the stock price using stock transaction history and text sentiments. This model improved the average root-mean-square error (RMSE) accuracy by 12.05. Lu et al. (2021) combined the outputs of the basic RNN and BERT-based models using an ensemble approach to solve the low-resource and sentiment polarity bias problems found in Aspect-based, Targeted Aspect and Multi-Entity Aspect-based sentiments using PEA. PEA provides significant improvements on all three tasks using only 20% of their training data. However, BiLSTM is a significantly slower model that takes longer to train and may require additional hardware like a GPU to reduce this time.

### Methodologies and process flow

**Figure 1:**
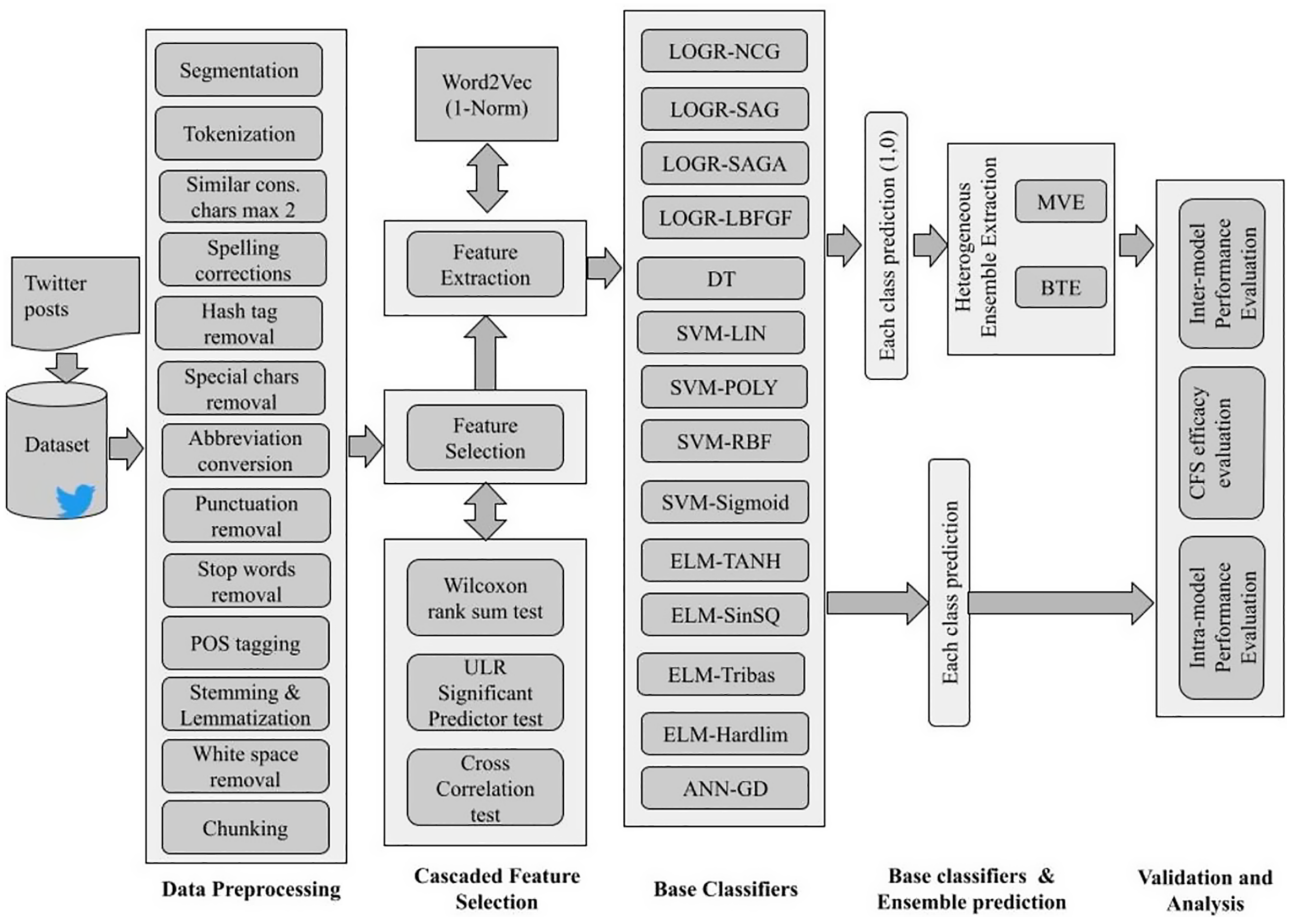
Semantic relational machine learning ensemble model architecture for sentiment classification.

The overall implementation schematic of SRML ensemble model is shown in Fig. 1.

As illustrated, Twitter review datasets are used that contain either two, three or five class polarity and different emotions. After pre-processing, it is observed that many words have no significance towards the sentiment contribution and hence can be removed. For this, we propose a novel Cascade Feature selection (CFS) technique that combines the “Wilcoxon rank sum test, ULR-based significant predictor test and cross-correlation test.” The strategic implementation of this feature selection method ensures optimal retention of only sentiment specific features. This is followed by word2vec feature extraction. However, the use of conventional word2vec will embed even those words which don’t have any significance towards sentiment. Therefore, in the proposed model word2vec is implemented along with CBOW configuration wherein it estimates the 1-norm and 2-norm features. The extracted features are then processed for weighing using SentiWordNet 3.0 for sentiment polarity.

**Figure 2:**
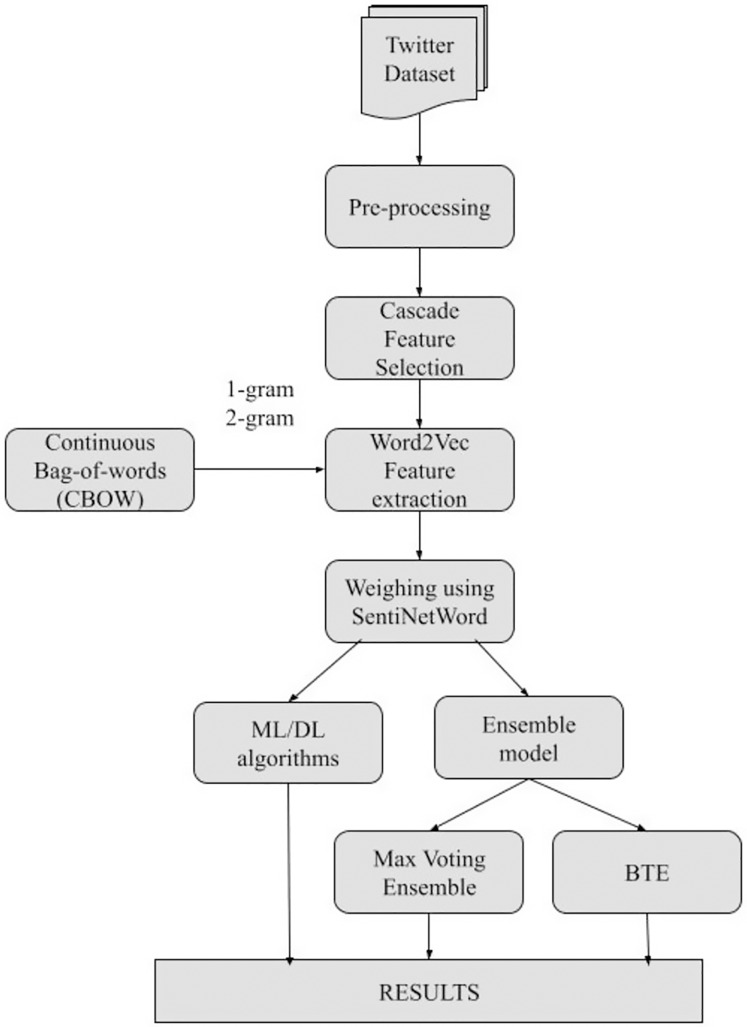
Stepwise flow-chart of SRML.

Overall, the SRML model is divided into five phases as shown in Fig. 2 flow-chart, and each phase is explained in brief below.

### Datasets

Our experiments were carried out on six different representative datasets with tweets on various subjects. The datasets used are: STS-Gold (termed as D1) (Saif et al., 2013), the Obama-McCain Debate (OMD) dataset (D2) (Diakopoulos & Shamma, 2010), the healthcare reform (HCR) dataset (D3) (Speriosu et al., 2011) and the SemEval2017 Task 4A (D4), 4B (D5) and 4C (D6) datasets (Rosenthal, Farra & Nakov, 2017). The datasets D1 to D3 are divided into three sets, training (60%), validation (20%) and testing (20%) and the hold-out technique was used to evaluate the predictive performance of the proposed method. The training and test set splits in datasets D4 to D4 as mentioned in Table 1 are used. Overall, the complete dataset statistics are shown in Table 1.

**Table 1:** Dataset statistics.

Dataset title and details	Class	Strongly negative	Negative	Neutral	Positive	Strongly positive	Total
D1: STS-GOLD (Saif et al., 2013)	2	–	1,402		632	–	2,034
D2: OMD (Diakopoulos & Shamma, 2010	2	–	1,196	–	710	–	1,906
D3: HCR (Speriosu et al., 2011)	2	–	1,369	–	539	–	1,908
D4: SemEval-2017, Task 4A Train (Rosenthal, Farra & Nakov, 2017)	3	–	7,840	22,591	19,902	–	50,333
D4: Test set	3		3,972	5,937	2,375	–	12,284
D5: SemEval-2017, Task 4B Train (Rosenthal, Farra & Nakov, 2017)	3	–	4,013	1,544	14,951	–	20,508
D5: Test set	3	–	3,722	–	2,463	–	6,185
D6: SemEval-2017, Task 4C Train (Rosenthal, Farra & Nakov, 2017)	5	299	3,398	12,993	12,922	1,020	30,632
D6: Test set	5	177	3,545	6,194	2,332	131	12,379

### Data acquisition and pre-processing phase

Majority of existing approaches have applied different review, feedback, or social media reaction datasets to perform two-class classification *i.e*., Positive and Negative sentiment also called polarity test using datasets with 3–4 sentiment labels or emotions. However, towards our goal to develop a robust expert system in-line with human behavior possessing multiple sentiments, class and complex datasets consisting of large-scale reviews from Twitter are used. These reviews are present in the row of the data while column name for each review is taken as Twitter-ID, sentiment, author, and the content. The raw input dataset is converted into a structured data-format. The sentence is first segmented and considering all the words present in the dataset, it is split into tokens. This is followed by removal of noise 1 *i.e*., #hashtags, @mentions, http//:URLs etc. (noise 1). Next, we remove any special unicode characters (noise 2), chat abbreviations conversions (noise 3), punctuation except ‘’ (noise 4) and stop words (noise 5). This is followed by POS tagging, stemming and lemmatization, white space removals and chunking leaving a heterogeneous dataset made up of integer variables (Twitter ID), characters, and strings (reviews or tweets) that are directly translated into a consistent numerical form to speed-up calculation. We apply normalization to each data element after getting the appropriate numerical outputs of each input feature variable, as explained in the next section.

### Data normalization phase

It is well known fact that data imbalance is a serious concern in classification or prediction systems, particularly large feature-based models. There’s a chance that the dataset under consideration has very minor limited features that indicate a sentiment. This can lead to classification bias, lowering overall prediction accuracy. Since the number of individuals and their information evaluated on Twitter is so large, the data they generate may be of various size and range. As a result, computing over such unstructured and broad-scaled data can lead to learning models to converge prematurely. It could potentially affect the overall correctness of the suggested model. Normalization is used to overcome this data imbalance problem. We employ the Min-Max technique, which normalizes input data in the range of 0 to 1, *i.e*., linearly transforms and translates input data-elements in the range of [0, 1]. The related normalized value *x*_*i'*_ in the range [0, 1] is transferred to each user feature *x* data element *x*_*i*_. We use Eq. (1) to calculate the normalized value(s) of the input data *x*_*i*_.

(1) $$Norm{\rm \; }\left( {{x_i}} \right) = {\rm \; }x_i^{\rm '} = {\rm \; }\displaystyle{{{x_i} - min\left( x \right)} \over {\max \left( x \right) - min\left( x \right)}}.$$

In Eq. (1), the data elements (user feature) *min(x)* and *max*(*x*) state the minimum and maximum values of *x* respectively.

### Feature selection phase

**Figure 3:**
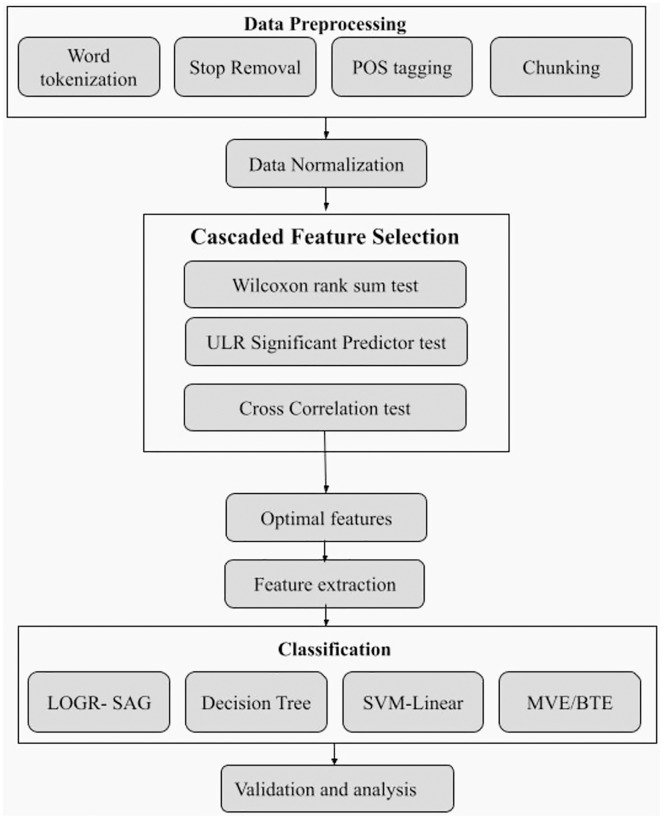
Schematic diagram of cascade feature selection approach.

In practical scenarios, a post or sentence can have certain words which don’t have any significance or relation with sentiments, and therefore removing such unwanted attributes is vital to enhance computational efficiency and accuracy. For *e.g*., consider a random tweet ‘*I really enjoyed the performance of the musician*’. Performing *n*-Gram (say 2-gram) decomposition on this post, we get ‘*I really*’, ‘*really enjoyed*’, etc. as bag-of-words (BoW). Here, ‘*really enjoyed*’ is connected to ‘*enjoy*’ sentiment, while ‘*I really*’ doesn’t have any relation to the sentiment. To remove such insignificant features from extracted data, a novel multi-phase cascade feature selection (CFS) as shown in Fig. 3 is proposed that uses three well-known statistical methods as explained below.

### Wilcoxon signed rank test (WRS)

The WRS test, also known as the rank test, is a non-parametric independent sample test that assesses the relationship between many variables and their impact on classification accuracy. The influence of word2vec-CBOW technique on sentiment categorization is calculated by considering the correlation between distinct feature values derived using this approach and their impact on sentiment categorization. In other words, the input vectors are categorized based on whether they are related to a user's emotion or even the likelihood of being a user’s sentiment. It demonstrates the relationship between each aspect and the sentiment. It uses independent and dependent variables, on which the correlation is estimated to discover the most significant variables with a strong relationship to the categorization result. The independent variable was designated as Twitter-ID, and the dependent variable was hypothesized to be its sentiment prediction or classification. Table 2 illustrates the algorithm used for finding the most optimal features using the WRS test. Using this method, *W* value is calculated from the pre-processed data partitioned into positive and negative opinions in the *corpus*. These are further used to form two matrices comprising each row with either positive or negative lexicon in association to each term. The difference between entries in both matrix columns with respect to feature *t* is ranked in ascending order and a sign of difference is applied. The sum of negative (*W−*) and positive (*W+*) ranks is found. For the signed rank obtained and based on the degree of probability (*p-*value), feature is optimal to positive set if absolute value of *W+* is greater than *W*. We calculate the *p-*value of each Twitter-ID in relation to the sentiment-proneness likelihood and illustrate how closely this is related to those attributes. Other features are deleted in favor of those with a stronger correlation (*e.g*., $\ge$0.5). As a result, WRS aids in managing uncertainty among all extracted characteristics and finds significant features by filtering out insignificant parts.

**Table 2:** Algorithm to find optimal features using Wilcoxon signed rank score.

**Input:** *Word vectors after SentiWordNet weighing*
**Output**: *Significant feature vectors*
**foreach** ${t_i}\; \in \; {w_l}$ *∈* ***begin***
$P,\; Q\; \leftarrow \; \emptyset$ //map empty sets used to store feature association frequency w.r.t. +ve and −ve sentiment lexicons
** foreach** ${p_j} \in \; {l_ + },\; \; {q_j} \in {l_ - }$ ***begin*** // for each +ve and −ve lexicon
$P \leftarrow {p_j}$ //add ${p_j}$ to $P$
$Q\; \leftarrow {q_j}$ //add ${q_j}$ to $Q$
Find W-value $\left( {P,\; Q} \right)$ //finding Wilcoxon signed rank score between $P,\; Q$
** if** (*W*-value doesn’t fit probability degree threshold *p-value*) ***begin***
Discard ${t_i}$ from the features list
** end**
** end**
**end**

### ULR assisted significant predictor test

In the same manner that the rank test analyses the inter-relationship between the independent and dependent variables, ULR does the same. This study investigates if a user’s Twitter ID, activity features, location features, and content features are significant predictors of sentiment-proneness on Twitter. ULR was applied to the selected characteristics from the previous selection phase (*i.e*., rank-sum selected features). The independent variable (*i.e*., user’s Twitter ID, activity features, location features, and content features) were used to compute the extent of variance (change %) in the dependent variable (sentiment prediction). The importance of a variable in terms of subsequent sentiment prediction is estimated using Eq. (2).

(2) $${\rm logit}\left[ {{\rm \pi }\left( {\rm x} \right)} \right] = {{\rm \alpha }_0} + {{\rm \alpha }_1}{\rm x}$$

The dependent (*i.e*., sentiment prediction) and independent (user’s Twitter ID and characteristics) variables are denoted by logit [ $\pi$(x)] and *x*, respectively. In Eq. (3), $\pi$ denotes the probability factor of significance for each category. Mathematically,

(3) $${\rm \pi }\left( {\rm x} \right) = \displaystyle{{{{\rm e}^{{{\rm \alpha }_0} + {{\rm \alpha }_1}{\rm X}}}} \over {1 + {{\rm e}^{{{\rm \alpha }_0} + {{\rm \alpha }_1}{\rm X}}}}}.$$

The value of the regression coefficient is used to estimate the significance level of each feature or text group (*i.e*., *p*-value). A feature element which has *p*-value >0.05 is considered significant for sentiment classification. Metrics having *p*-value <0.05 are dropped.

### Cross-correlation test

In this method, a cross-correlation test is run on a ULR filtered feature-set containing the user’s Twitter ID, activity data, location information, and content features, using the Pearson correlation coefficient which is a representative way to measure similarity. It is the ratio of the covariance to the standard deviation. It has relatively high requirements on the data. The Euclidean distance (the distance between vectors) is generally used to measure the similarity of vectors, but the Euclidean distance cannot consider the difference of values between different variables (Liu et al., 2022). The Pearson correlation coefficient can be calculated using Eq. (4).

(4) $$P\; = \; \displaystyle{{cov\; \left( {X,\; Y} \right)} \over {\sigma \left( X \right).\sigma \left( Y \right)}}$$

User attributes having a correlation coefficient larger than 0.5 (*p* > 0.5) are chosen as the final feature vectors for further sentiment classification.

### Feature extraction phase

Considering heterogeneity of the datasets, the use of conventional feature extraction methods such as information gain etc. might lead to inaccuracy. We propose a combination of word2vec-CBOW method which retrieves 1-and 2-gram words. Dictionary is used to estimate value of these words. In addition, the use of word2vec enables extraction of semantic features which can exploit intent or aspect information to perform further sentiment classification. The algorithms considered make use of tokenized words and after matching dictionary values, feature value is estimated for each feature (1-gram and 2-gram words). Thus, to estimate feature value of each tweet or review, addition of all vectors is performed in a word or a particular Twitter post. The extracted features are then processed for weighing using SentiWordNet 3.0 (Baccianella, Andrea & Fabrizio, 2010). This helps in automatic annotation of all the WordNet synsets according to their degree of ‘positivity’, ‘negativity’ and ‘neutrality’. We thus get any of the three numerical scores *i.e*., Pos(s), Neg(s) and Obj(s) for neutral. Each of three scores ranges in the interval [0.0,1.0] and their sum is 1.0 for each synset. If a synset has non-zero score for all three categories, it means that the corresponding tweet has each of the three sentiment-related properties to some degree. If it is zero for a particular category, then the tweet is very clearly positive or negative accordingly.

### Ensemble classification

To design a classifier ensemble, 14 individual classifiers or learners with different kernel functions are implemented both independently and in an ensemble. The individual classifiers are logistic regression (under this, implemented algorithms are Newton-Conjugate Gradient, Stochastic Average Gradient, SAGA- A fast incremental Gradient method with support for Non-Strongly Convex Composite Objectives and LBFGS- the Limited Memory Broyden Fletcher Goldfarb Shanno algorithm), Decision Tree Classifier, support vector machines (under this Linear, Polynomial, Radial Basis Function and Sigmoid activation function are implemented), extreme learning machines (with Tanh, SinSQ, Tribas-Triangular basis transfer function and Hardlim-Hard limit transfer function) and Artificial Neural Networks MLP (Multi-Layer Perceptron) Gradient Descent. Since the proposed ensemble comprises machine learning algorithms from different paradigms, it is termed as a heterogeneous ensemble learning (HEL) model. Majority voting ensemble (MVE) is the ensemble technique and we also choose a best trained ensemble (BTE) which is explained below.

### Decision tree

**Figure 4:**
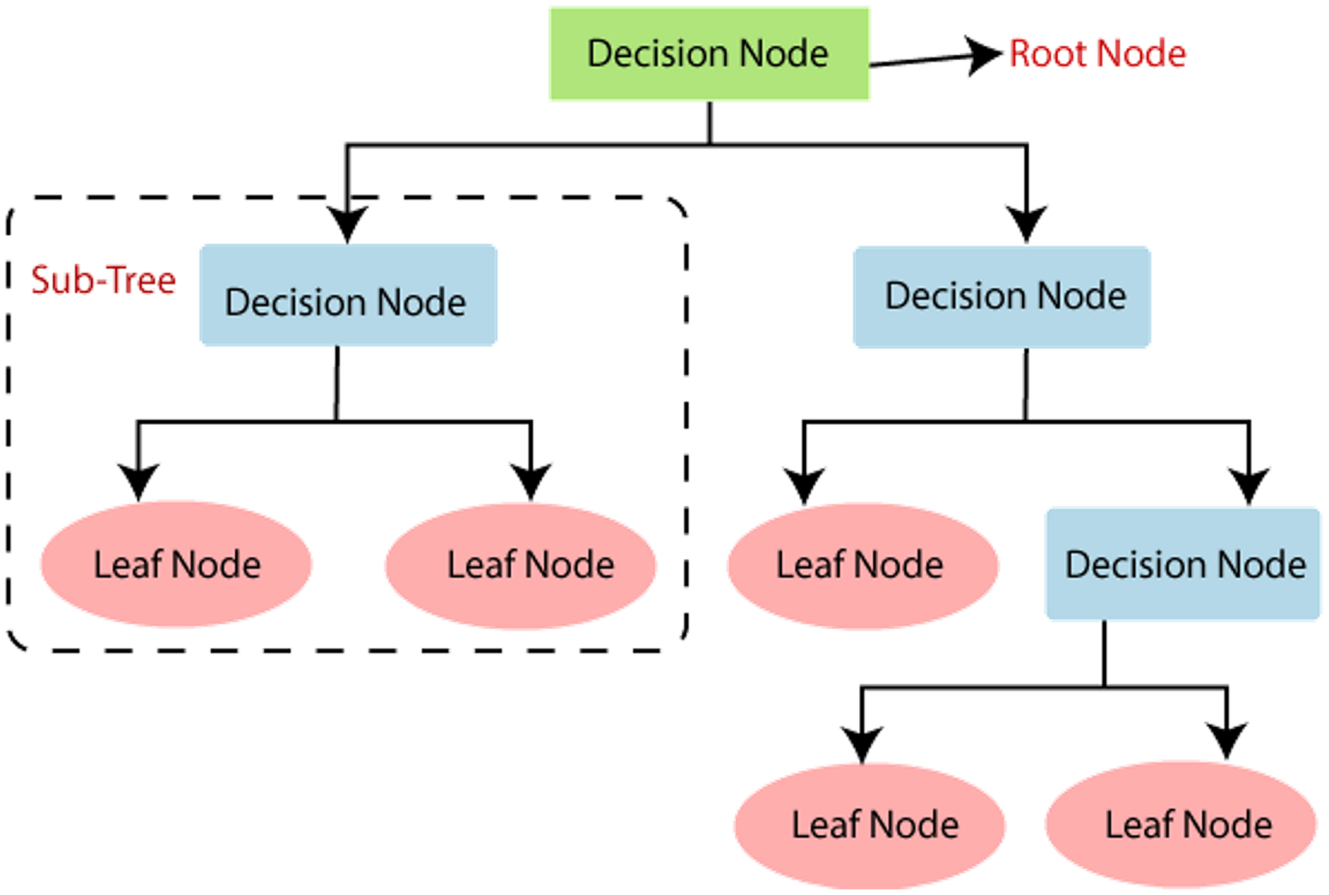
Decision tree used for sentiment classification.

The C5.0 DT classifier model is used which carries out recursive partitioning on extracted datasets to predict job for a specified user’s input. Association rule mining is used to split the feature vector at each node of the tree to form different branches as shown in Fig. 4.

### Logistic regression (LOGR)

Regression is applied on the independent and dependent variables to perform classification of the dependent variable. In this research, LOGR defines a prediction scheme that checks the semantic correlation between the sentiment and tweet post. Mathematically, LOGR can be calculated as in Eq. (5).

(5) $$logit\left[ {\pi \left( x \right)} \right] = {\beta _0} + {\beta _1}{X_1} + {\beta _2}{X_2} + \ldots \ldots . + {\beta _m}{X_m}$$

LOGR returns ${\rm \pi }\left( {\rm x} \right)$ as Eq. (6):

(6) $$\pi \left( x \right) = \displaystyle{{{e^{{\beta _0} + {\beta _1}{X_1} + {\beta _2}{X_2} + \ldots \ldots . + {\beta _m}{X_m}}}} \over {1 + {e^{{\beta _0} + {\beta _1}{X_1} + {\beta _2}{X_2} + \ldots \ldots . + {\beta _m}{X_m}}}}}.$$

In this work, Logistic regression with Newton-Conjugate Gradient, Stochastic Average Gradient, SAGA- a fast incremental Gradient method with support for Non-Strongly Convex Composite Objectives and LBFGS- Limited Memory Broyden Fletcher Goldfarb Shanno algorithm are implemented.

### Support vector machine (SVM)

**Figure 5:**
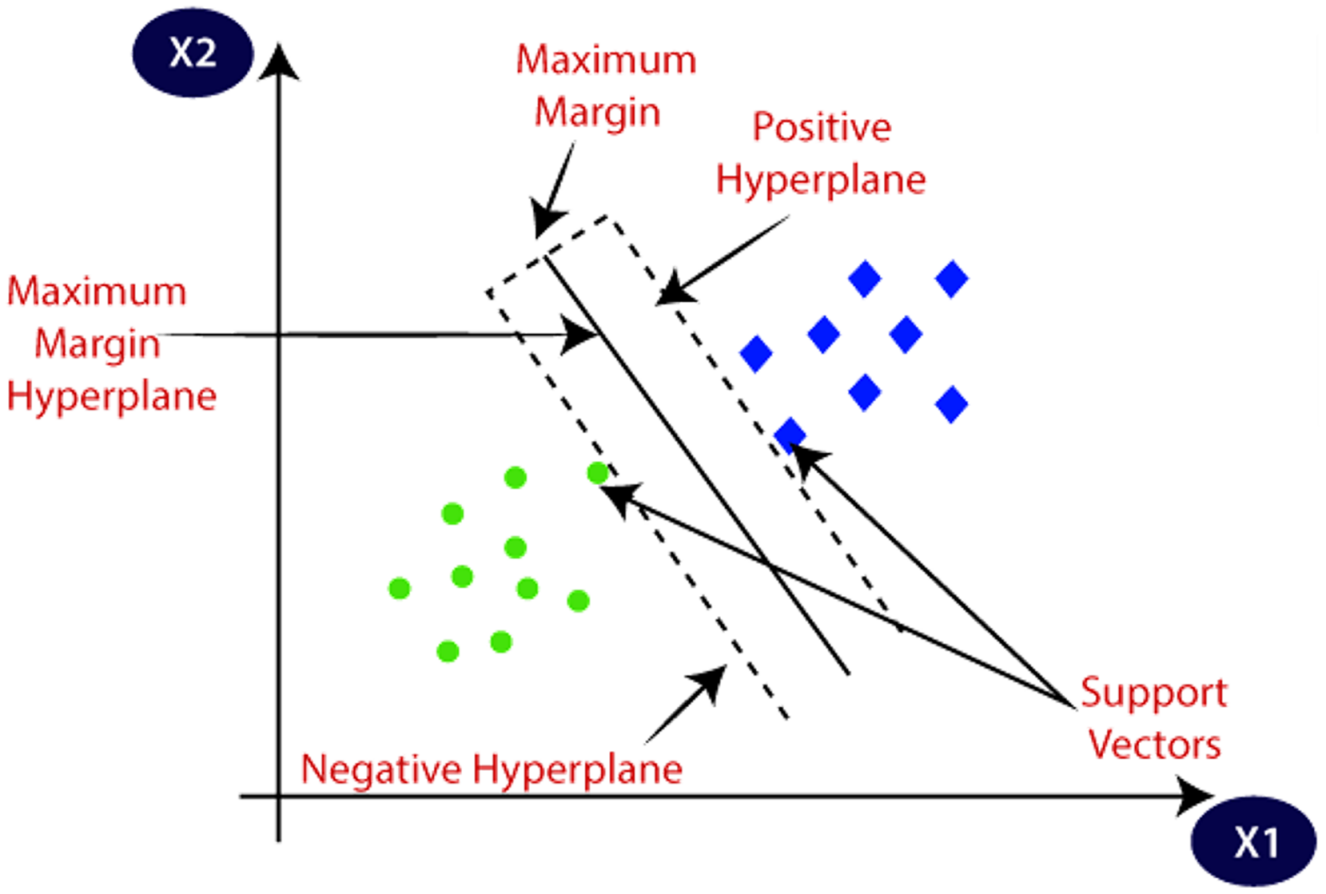
Illustration of support vector machine.

SVM exploits the pattern of the data and functions as a non-probabilistic binary linear classifier as shown in Fig. 5.

To predict the class, SVM applies the function in Eq. (7) as:

(7) $${Y}^{\prime} = w * \phi \left( x \right) + b$$

In Eq. (8), ${\rm \; }{Y}^{\prime}$ is retrieved by reducing the risk of regression.

(8) $${R_{reg}}\left( {{Y}^{\prime}} \right) = C * \mathop \sum \nolimits_{i = 0}^l \gamma \left( {Y_i^{\rm '} - {Y_i}} \right) + \displaystyle{1 \over 2} * {\Vert w\Vert^2}$$

where,

(9) $$w = \mathop \sum \nolimits_{j = 1}^l \left( {{\alpha _j} - \alpha _j^{\rm *}} \right)\phi \left( {{x_j}} \right)$$

In above Eq. (9), the parameters $\alpha$ and ${\alpha ^{\rm *}}$ state the relaxation parameter called Lagrange multiplier. The output obtained is,

(10) $${Y}^{\prime} = \mathop \sum \nolimits_{j = 1}^l \left( {{\alpha _j} - \alpha _j^{\rm *}} \right)\phi \left( {{x_j}} \right) * \phi \left( x \right) + b$$

(11) $${Y}^{\prime} = \mathop \sum \nolimits_{j = 1}^l \left( {{\alpha _j} - \alpha _j^{\rm *}} \right) * K\left( {{x_j},x} \right) + b$$

In Eqs. (10) and (11), $K\left( {{x_j},x} \right)$ states the kernel function. In this work, SVM algorithm with Linear, Polynomial, Radial Basis Function and Sigmoid activation function are implemented.

### Artificial neural network (ANN)

**Figure 6:**
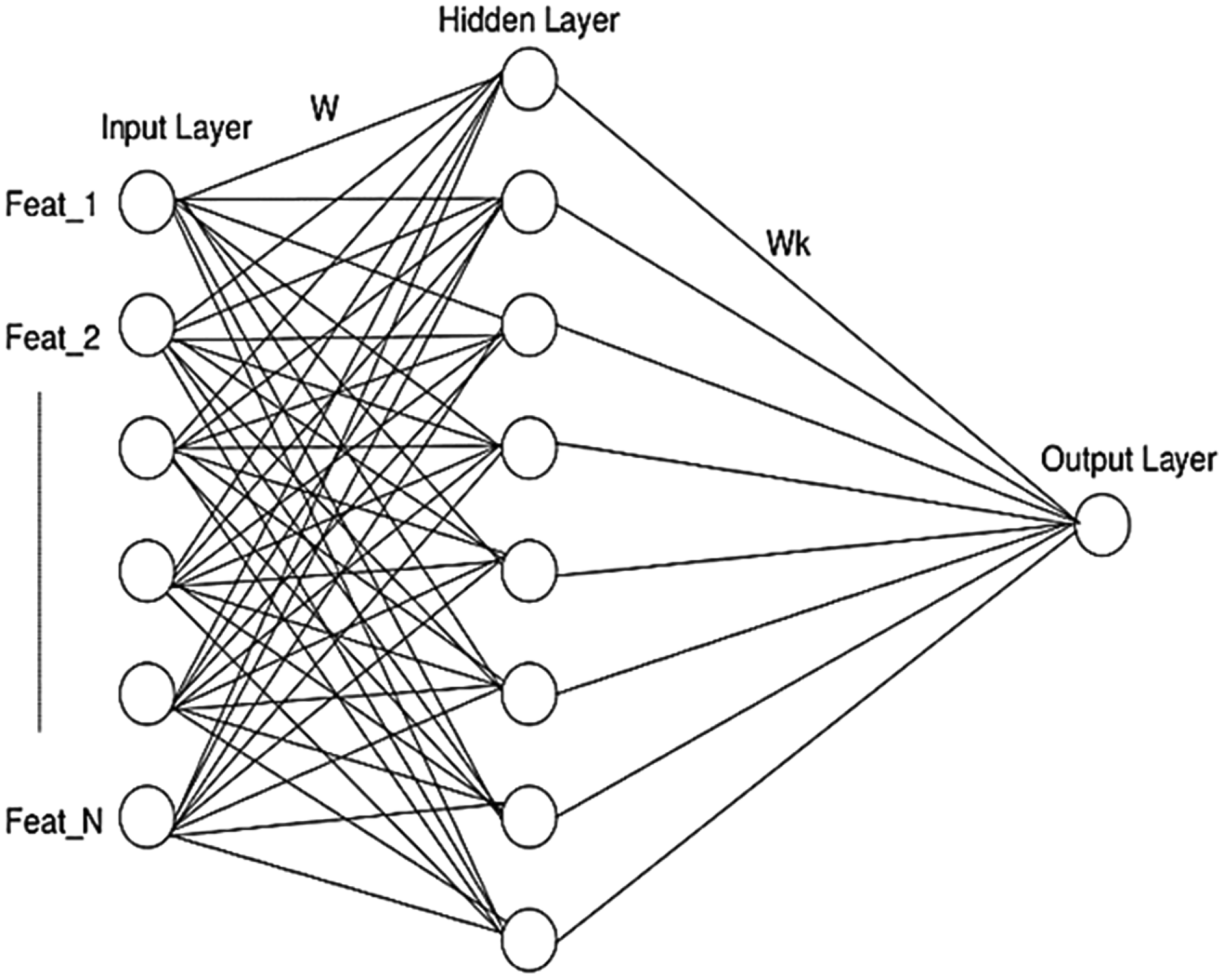
ANN architecture used in SRML.

A conventional architecture of ANN comprising three layers- input, hidden and output layer is illustrated in Fig. 6. The final feature vector pertaining to each post is fed as input and classified into different sentiment classes which results in an output on account of the hidden layer sigmoid function applied.

With the output of the input layer (input of the hidden layer ${\rm \; }{I_h}$), the output at ${O_h}$ will be as in Eq. (12).

(12) $${O_h} = \displaystyle{1 \over {1 + {e^{ - {I_h}}}}}$$

And the final output after ANN learning will be as in Eq. (13).

(13) $${O_o} = \displaystyle{1 \over {1 + {e^{ - {O_i}}}}}.$$

To perform accurate classification ANN reduces error value iteratively. Mathematically, the error function is obtained as in Eq. (14).

(14) $$MSE = {\rm \; }\displaystyle{1 \over n}\mathop \sum \nolimits_{i = 1}^n {\left( {{y_i}^{\rm '} - {y_i}} \right)^2}.$$

In this work, ANN MLP with gradient descent is implemented.

### Extreme learning machine (ELM)

In most ANN variants, the predominant issue is local minima and convergence that becomes severe in case of large-scale training dataset and affects overall learning and classification efficiency. To address this issue, ELM with three different kernel functions *i.e*., linear, polynomial and RBF are proposed as base classifiers for sentiment classification.

Output of the proposed ELM base classifier is given by Eq. (15) as:

(15) $$y\left( {t + k} \right) = f\left( X \right) = \mathop \sum \nolimits_{i = 1}^L {\beta _i}G\left( {{a_i},{b_i},X} \right)$$

In this work, ELM with Tanh, SinSQ, Tribas and Hardlim transfer functions are implemented.

### Heterogeneous ensemble learning (HEL)

Ensemble learning is a well-defined and strategically implemented machine learning technique which combines independent classifiers (base learners) to perform classification. Ensemble technique is often used to boost up the performance of slow base learners and to improve overall accuracy. In the proposed method, first the sentiment score (*SS*) of the tweet is calculated using the algorithm as shown in Table 3. The training data consisting of a sequence of test tweets was used to train the system. The sentiment (Positive/Negative) of each tweet in this test tweet is determined by each base classifier in the ensemble. In addition, each base classifier’s predictive performance was evaluated using the hold out technique on the validation data and the best hyper-parameters tuned models were used on the testing data or test tweets. The next step is to figure out how likely each tweet is to be positive or negative. After allocating this probability, we use the ensemble technique to provide weight to each classifier depending on its accuracy. Finally, the computer generates the tweet’s positive and negative score based on each classifier’s prediction.

**Table 3:** Algorithm for calculating sentiment score of a tweet.

**Input:** $Testtweet\left( {TT} \right)$;
**Output**: $Sentiscore\left( {SS} \right)$
**foreach** ${T_i}$ in $TT$ **do** // ${T_i}$ is ${i^{th}}$tweet
$P{C_i} = 0$ // *i*^*th*^ positive count
$N{C_i} = 0$ // *i*^*th*^ negative count
** foreach** classifier ${C_{ i}}{\; }$in ensemble **do**
** if** ${C_{ i}}{\; }$predicts positive **then**
$P{C_{ i}}{\; } = {\; } + l$;
** end**
** else**
$N{C_{ i}}{\; } = {\; } + l$;
** end**
**end**
$Prob\left( {Positiv{e_i}} \right) = {\rm \; }\displaystyle{{P{C_i}} \over {P{C_i} + {\rm \; }N{C_i}}}$
$Prob\left( {Negativ{e_i}} \right) = {\rm \; }\displaystyle{{N{C_i}} \over {P{C_i} + {\rm \; }N{C_i}}}$
**end**
**for** each classifier ${C_{ i}}$ in ensemble **do**
$Weigh{t_{{C_i}}} = {\rm \; }\displaystyle{{ac{c_{{C_i}}}} \over {\mathop \sum \nolimits_{j = 1}^n ac{c_{{C_j}}}}}$
**end**
**foreach** ${T_{ i}}$ in *TT* **do**
$P{S_{i}} = 0$ // *i*^*th*^ positive score
$N{S_{i}} = 0$ // *i*^*th*^ negative score
** foreach** classifier ${C_{ i}}$ in ensemble **do**
** if** ${C_{ i}}{\; }$predicts positive **then**
$P{S_{i\; }} =$ $Weigh{t_{{C_i}}}\; + Prob\left( {Positiv{e_i}} \right)$;
** end**
** else**
$N{S_{i\; }} = Weigh{t_{{C_i}}}\; + Prob\left( {Negativ{e_i}} \right)$;
** end**
** end**
** return** $P{S_i}$, $N{S_i}$
**end**

The sentiment of the tweet is predicted by the algorithm as mentioned in Table 4. The positive and negative score of the tweet is used as inputs to this algorithm. If a tweet’s positive score exceeds its negative score, the sentiment of that tweet is considered positive and vice versa. Finally, if a tweet’s positive and negative scores are equal, the system computes the cosine similarity of that tweet to all other tweets in the testing data and determines which tweet is the most similar. The positive and negative score of the identified tweet is then calculated. If the positive score exceeds the negative score, the tweet is considered positive; otherwise, it is considered negative.

**Table 4:** Algorithm for sentiment prediction of a tweet in ensemble model.

**Input:** ${T_{i\; }},\; P{S_{i\; }},\; \; N{S_{i\; }}$
**Output:** Sentiment (*S*)
**if** $P{S_{i\; }} > \; N{S_{i\; }}$ then
*S* = ‘*positive*’;
**else**
** if** $N{S_{i\; }} > \; P{S_i}$ **then**
*S* = ‘*negative*’;
** else**
Calculate cosine similarity of ${T_i}$ with all other tweets in test_data
using distance calculation formulation
Find most similar tweet of ${T_i}$ say ${T_j}$
Calculate $P{S_i}$ and $N{S_{i\; }}$ of ${T_j}$ using Algorithm in Table 3
Use maximum voting
** if** $P{S_i}{\rm \; } > N{S_{i\; }}$ **then**
*S* = ‘*positive*’;
** else**
*S* = ‘*negative*’;
** end**
** end**
**end**

‘Cosine similarity’ is a distance measurement that compares the similarity of two tweets that was used. Equation (16) states the formula for computing cosine similarity.

(16) $$Cos\left( {{T_1},{\rm \; }{T_2}} \right) = {\rm \; }\displaystyle{{{T_1} * {T_2}} \over {||{T_1}\left| {\left|\ *\ \right|\left| {{T_2}} \right|} \right|}}$$

where, ${T_1}and{\rm \; }{T_2}$ represent vectors and output value one represents high similarity.

In this work, majority voting ensemble technique is used and results are captured using two ensemble models as explained below.

### Majority voting based ensemble (MVE) for sentiment classification

Here, all classifiers are executed over the same dataset (feature vector) which classifies/labels each tweet or review (related feature) as a specific type based on sentiment score using Algorithm1. For example, for two class polarity test (negative and positive sentiment) a classifier predicts a score for each tweet as Positive (say, 1) or Negative (say, 0). Since our data consists of multiple emotions, the classifier finally predicts and labels the output based on feature’s relative tilt towards positive or negative score. The MVE uses ‘maximum voting’ to find this sentiment. In other words, a sentiment with the highest number of 1’s is predicted as ‘positive’, else it is ‘negative’.

### Best trained ensemble (BTE) for sentiment classification

BTE typically identifies the best performing classifiers among the individual classifiers and chooses the one or multiple with the highest prediction accuracy to perform classification. In this work, fourteen individual classifiers have been used and performance (here, prediction accuracy) of each classifier is obtained. Majority voting is used and in the proposed ensemble model, only those individual classifiers which provide an average accuracy of ≥70% are considered to constitute the BTE ensemble. The laggards are dropped.

### Performance metrics

For assessing the efficacy of the Cascade Feature selection model, optimal features are selected from all the six datasets D1 to D6 using the proposed CFS approach and compared with the existing count vectorization method. The reduced features are validated on four classifiers namely LOGR-SAG, ANN-GD, SVM-Linear and the Majority Voting Ensemble (MVE). The accuracy of each of the classifier is calculated using Eq. (17).

(17) $${\rm Accuracy\; (Acc)} =\displaystyle{{\left( {TN{\rm \; } + TP} \right)} \over {\left( {TN + FN + FP + TP} \right)}} \times 100.$$

For assessing the performance of the CFS-based SRML model, two distinct assessments *i.e*., the Intra-model and Inter-model assessment is carried out. Apart from accuracy, precision as per Eq. (18), recall as per Eq. (19) and *F1*-score as per Eq. (20) are the standard performance metrics used for evaluation based on True Positive (*TP*), True Negative (*TN*), False Positive (*FP*) and False Negative (*FN*) values.

(18) $${{\rm Precision} \;(Pre)}=\displaystyle{{TP} \over {\left( {TP + FP} \right)}}\times 100$$

(19) $${{\rm Recall}\; (Rec)}=\displaystyle{{TP} \over {\left( {{\rm TP} + {\rm FN}} \right)}}\times 100$$

(20) $${F1{-}{\rm Score}}=2\times \displaystyle{{precision \times recall} \over {precision + recall}}.$$

For dataset D4, we calculate average *F1*-score over positive and negative class as $F{1^{PN}}$, excluding neutral class using Eq. (21).

(21) $${{\rm Average}\;{F1}{(F{1^{PN}})}}=\displaystyle{1 \over 2}\; \left( {F{1^{Positive}} + F{1^{Negative}}} \right)\times 100$$

For datasets D4 and D5, we calculate the average recall (*AveRec*) which is calculated by considering the average of positive, negative and neutral recall values as per Eq. (22).

(22) $${{\rm Average \; Recall}\; (AveRec)}=\displaystyle{1 \over 3}\; \left( {Re{c^{Positive}} + Re{c^{Negative}} + Re{c^{Neutral}}} \right)\times 100$$

For Inter-model comparison on dataset D6, we calculate the Macro average mean absolute error $\left( {MA{E^M}} \right)$ as the classification measure (Rozental & Fleischer, 2017) using Eq. (23).

(23) $$\eqalign{& {\rm Macro\; Average\; Mean\; Absolute\; Error\; }\left( {{\rm MA}{{\rm E}^{\rm M}}} \right) \cr& \left( {h,{T_e}} \right) = \displaystyle{1 \over {\left| C \right|}}\mathop \sum \nolimits_{j = 1}^{\left| C \right|} \displaystyle{1 \over {\left| {{T_{{e_j}}}} \right|}}\mathop \sum \nolimits_{{x_i} \in {T_{{e_j}}}} \left| {h\left( {{x_i}} \right) - {y_i}} \right| } $$

where ${y_i}$ denotes the true label of ${x_i}$, and $h\left( {{x_i}} \right)$ is its predicted label, ${T_{{e_j}}}$ represents the set of test documents whose true class is ${c_j}$. $\left| {h({X_i}) - {y_i}} \right|$ represents the distance between classes $h({x_i})$ and ${y_i}$. For example, we consider the distance between highly positive and negative is three.

In Intra-model assessment, relative performance parameters mentioned above are compared for each individual classifier and the ensemble classifier models. In Inter-model assessment, we compare and present the efficacy of the proposed CFS-based SRML model against state-of-the-art methods that have used various Machine Learning and Deep Learning paradigms on similar datasets.

## Results and discussion

The implementation of the proposed model, training and testing is carried out on Windows 64-bit Operating system with Intel Core i7, 16GB memory system. The models were developed using Python and NLTK tool kit.

### CFS feature selection assessment

**Figure 7:**
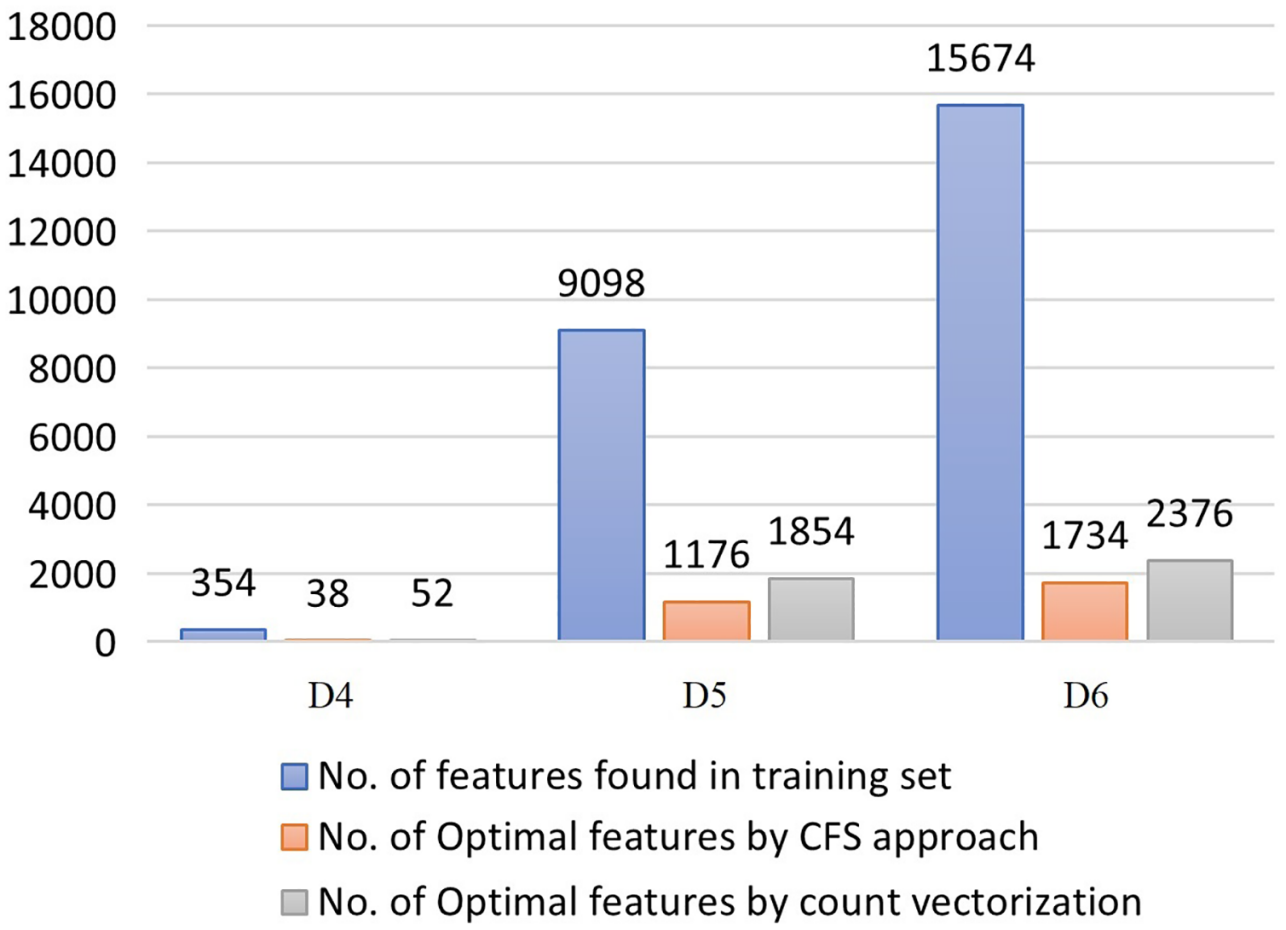
Optimal feature selection statistics.

The process of selecting the reduced set of attributes using the Cascade feature selection and count vectorizer (CV) approach for all the three SemEval 2017 datasets is depicted in Fig. 7.

Further, the reduced features are validated using three individual classifiers, the LOGR-SAG, ANN-GD, SVM-Linear and their ensemble using majority voting technique (MVE). Results are shown in Table 5.

**Table 5:** Classifier accuracy comparison using the CFS approach.

Dataset	STS-Gold		OMD		HCR		SemEval Task 4A		SemEval Task 4B		SemEval Task 4C	
Method	Acc. (%) with CV	Acc. (%) with CFS	Acc. (%) with CV	Acc. (%) with CFS	Acc. (%) with CV	Acc. (%) with CFS	Acc. (%) with CV	Acc. (%) with CFS	Acc. (%) with CV	Acc. (%) with CFS	Acc. (%) with CV	Acc. (%) with CFS
LOGR-SAG	81.92	83.01	82.28	83.29	78.39	80.89	65.43	69.52	75.72	76.98	73.65	76.32
ANN-GD	85.27	87.82	84.58	85.18	80.76	81.28	56.73	60.13	73.87	76.76	72.86	74.38
SVM-Lin	81.02	82.37	78.92	81.73	78.27	80.29	64.44	69.32	72.19	75.32	69.59	73.27
MVE	82.73	83.69	81.29	83.23	79.39	81.94	69.87	73.32	76.87	78.29	74.54	77.89

Results from the experimental study on all the datasets depict that CFS can support in attaining a higher classification accuracy with up to 50% lesser features compared to count vectorizer approach. The approach retains the overall performance of the classifiers even under minimal number of features selected. The results of the Majority voting ensemble are higher compared to all other individual classifiers, signifying CFS approach as an optimal feature selection strategy and that can be incorporated in the proposed SRML model. We then go on to evaluate if it can help in improving the overall performance of the SRML model compared to current state-of-the-art models.

### Intra-model performance assessment

Tables 6–8 presents the results of all individual classifiers and the two heterogeneous ensemble techniques proposed on the datasets STS-Gold (D1), OMD (D2) and HCR (D3) respectively. Tables 9–11 present the results achieved by individual classifiers and the heterogeneous ensemble models on the SemEval 2017 Task 4A, 4B and 4C respectively for sentiment classification.

**Table 6:** Experimental results of individual classifiers and ensemble model on dataset D1 (Best results in bold).

Techniques	Acc. (%)	Positive class			Negative class			Average
		Prec (%)	Rec (%)	F1 (%)	Prec (%)	Rec (%)	F1 (%)	F1 (%)
Individual classifiers								
LOGR-NCG	73.28	73.45	78.38	75.83	74.04	77.89	75.92	75.88
LOGR-SAG	83.01	76.59	72.37	74.42	76.35	73.55	74.92	74.67
LOGR-SAGA	75.87	73.79	74.32	74.05	74.79	73.32	74.05	74.05
LOGR-LBFGS	79.49	76.39	75.21	75.80	76.33	76.11	76.22	76.01
DT	69.98	63.27	62.89	63.08	62.89	63.93	63.41	63.25
SVM-Lin	82.37	81.28	76.38	78.75	81.78	77.83	79.76	79.26
SVM-Poly	85.37	82.13	79.82	80.96	81.69	79.21	80.43	80.70
SVM-RBF	83.49	**83.98**	**80.18**	**82.04**	**84.03**	**80.45**	**82.20**	**82.12**
SVM-Sig	**87.82**	82.92	76.39	79.52	83.21	75.98	79.43	79.48
ELM-T	73.29	73.48	69.82	71.60	73.41	69.33	71.31	71.46
ELM-SIN	75.39	76.32	70.85	73.48	75.86	71.37	73.55	73.52
ELM-TRI	74.26	69.48	67.82	68.64	68.98	67.98	68.48	68.56
ELM-HL	73.47	71.28	65.83	68.45	71.58	66.23	68.80	68.63
ANN-GD	**87.82**	82.37	79.94	81.14	81.66	80.31	80.98	81.06
Ensemble classifiers								
MVE	83.69	85.25	82.39	83.80	85.75	82.74	84.22	84.01
BTE	**85.83**	**86.29**	**85.29**	**85.79**	**86.61**	**86.21**	**86.41**	**86.10**

**Table 7:** Experimental results of individual classifiers and ensemble model on dataset D2 (Best results in bold).

Techniques	Acc (%)	Positive class			Negative class			Average
		Prec (%)	Rec (%)	F1 (%)	Prec (%)	Rec (%)	F1 (%)	F1 (%)
Individual classifiers								
LOGR-NCG	83.93	73.29	75.45	74.35	74.32	74.95	74.63	74.49
LOGR-SAG	83.29	74.83	76.39	75.60	74.21	75.99	75.09	75.35
LOGR-SAGA	85.12	72.39	72.36	72.37	73.09	72.68	72.88	72.63
LOGR-LBFGS	82.8	76.52	75.48	76.00	76.96	74.98	75.96	75.98
DT	71.93	70.12	74.29	72.14	70.68	75.32	72.93	72.54
SVM-Lin	81.79	82.14	84.28	83.20	83.11	**85.22**	**84.15**	83.68
SVM-Poly	83.74	**84.18**	82.19	83.17	**84.23**	83.10	83.66	83.42
SVM-RBF	84.74	80.97	81.87	81.42	81.08	82.23	81.65	81.54
SVM-Sig	**85.96**	83.91	83.5	83.37	84.21	83.86	84.03	**83.70**
ELM-T	78.39	73.29	71.75	72.51	74.19	72.35	73.26	72.89
ELM-SIN	76.82	75.83	70.23	72.92	76.33	71.32	73.74	73.33
ELM-TRI	70.27	71.29	74.39	72.81	71.29	74.39	72.81	72.81
ELM-HL	72.29	68.56	71.28	69.89	68.63	71.81	70.18	70.04
ANN-GD	85.18	82.94	**84.39**	**83.66**	83.01	84.39	83.69	83.68
Ensemble classifiers								
MVE	83.23	84.27	81.09	82.65	83.77	80.96	82.34	82.50
BTE	**87.82**	**84.98**	**85.01**	**84.99**	**84.90**	**85.11**	**85.00**	**85.00**

**Table 8:** Experimental results of individual classifiers and ensemble model on dataset D3 (Best results in bold).

Techniques	Acc (%)	Positive class			Negative class			Average
		Prec (%)	Rec (%)	F1 (%)	Prec (%)	Rec (%)	F1 (%)	F1 (%)
Individual classifiers								
LOGR-NCG	83.32	74.38	74.29	74.33	73.33	74.92	74.12	74.23
LOGR-SAG	80.89	76.39	79.45	77.89	76.53	79.41	77.94	77.92
LOGR-SAGA	83.23	75.43	80.13	77.71	76.13	80.35	78.18	77.95
LOGR-LBFGS	**84.15**	78.76	78.92	78.84	78.99	79.22	79.10	78.97
DT	78.29	73.29	74.57	73.92	73.62	74.76	74.19	74.06
SVM-Lin	80.29	84.39	81.29	**82.81**	84.34	81.65	**82.97**	**82.89**
SVM-Poly	81.23	82.17	**83.33**	82.75	82.37	83.45	82.91	82.83
SVM-RBF	80.82	81.27	82.97	82.11	81.27	82.97	82.11	82.11
SVM-Sig	80.02	**84.39**	80.19	82.24	**84.39**	80.19	82.24	82.24
ELM-T	78.03	73.34	74.39	73.86	73.14	73.48	73.31	73.59
ELM-SIN	79.05	76.29	72.39	74.29	76.21	72.23	74.17	74.23
ELM-TRI	79.36	72.39	74.28	73.32	72.31	74.31	73.30	73.31
ELM-HL	76.04	74.12	71.12	72.59	74.11	71.42	72.74	72.67
ANN-GD	81.28	81.28	83.12	82.19	81.33	**83.52**	82.41	82.30
Ensemble classifiers								
MVE	81.94	82.19	81.11	81.65	82.23	81.21	81.72	81.69
BTE	**86.09**	**85.16**	**84.19**	**84.67**	**85.68**	**84.39**	**85.03**	**84.85**

**Table 9:** Experimental results of individual classifiers and ensemble model on dataset D4 (Best results in bold).

Techniques	Acc (%)	AvgRec (%)	${F}{1^{{PN}}}$(%)
Individual classifiers			
LOGR-NCG	69.78	65.92	67.04
LOGR-SAG	68.98	65.43	65.29
LOGR-SAGA	71.09	64.28	65.08
LOGR-LBFGS	68.93	64.35	64.96
DT	65.09	64.29	61.67
SVM-Lin	68.42	62.19	63.22
SVM-Poly	68.02	59.98	64.19
SVM-RBF	65.94	67.23	64.85
SVM-Sig	67.82	66.93	64.05
ELM-T	56.82	59.88	58.12
ELM-SIN	59.82	57.29	57.42
ELM-TRI	59.02	54.71	58.27
ELM-HL	60.03	58.37	59.99
ANN-GD	**71.98**	**67.55**	**69.32**
Heterogeneous ensemble learning (HEL) models			
MVE	71.35	67.82	69.49
BTE	**74.87**	**71.28**	**72.71**

**Table 10:** Experimental results of individual classifiers and ensemble model on dataset D5 (Best results in bold).

Techniques	Acc (%)	Positive class			Negative class			Average
		**Prec (%)**	**Rec (%)**	**F1 (%)**	**Prec (%)**	**Rec (%)**	**F1 (%)**	**F1 (%)**
Individual classifiers								
LOGR-NCG	87.97	85.39	84.39	84.89	87.37	84.29	85.8	85.34
LOGR-SAG	85.59	83.49	82.98	83.23	80.09	83.29	81.66	82.45
LOGR-SAGA	88.89	83.29	83.98	83.63	82.89	84.2	83.54	83.59
LOGR-LBFGS	83.89	81.29	83.29	82.28	82.99	85.3	84.13	83.20
DT	74.88	75.98	73.49	74.71	75.92	72.39	74.11	74.41
SVM-Lin	81.29	80.39	79.29	79.84	82.39	80.44	81.29	80.56
SVM-Poly	82.39	82.78	83.49	83.13	81.39	80.27	80.83	81.98
SVM-RBF	81.98	80.21	79.49	79.85	83.2	80.29	81.72	80.78
SVM-Sig	80.29	81.74	80.38	81.05	81.29	82.87	82.07	81.56
ELM-T	79.39	80.28	78.49	79.37	81.29	79.4	80.33	79.85
ELM-SIN	82.1	83.8	81.71	82.74	83.87	82.74	83.3	83.02
ELM-TRI	82.87	83.75	84.55	84.15	82.58	84.29	83.43	83.79
ELM-HL	83.71	82.48	83.87	83.17	82.68	81.28	81.97	82.57
ANN-GD	**89.47**	**87.77**	**90.21**	**88.97**	**91.29**	**89.76**	**90.52**	**89.75**
Ensemble classifiers								
MVE = BTE	**92.83**	**95.39**	**93.89**	**94.63**	**94.93**	**94.78**	**94.85**	**94.74**

**Table 11:** Experimental results of individual classifiers and ensemble model on dataset D6 (Best results in bold).

Techniques	Acc (%)	${MA}{{E}^{{M}}}$
Individual classifiers		
LOGR-NCG	73.23	0.713
LOGR-SAG	71.27	0.727
LOGR-SAGA	72.48	0.718
LOGR-LBFGS	73.23	0.734
DT	72.65	0.824
SVM-Lin	70.58	0.829
SVM-Poly	67.43	0.798
SVM-RBF	61.38	0.812
SVM-Sig	62.87	0.827
ELM-T	61.78	0.783
ELM-SIN	65.87	0.718
ELM-TRI	63.98	0.698
ELM-HL	62.98	0.729
ANN-GD	**74.34**	**0.687**
Heterogeneous ensemble learning (HEL) models		
MVE	74.88	0.565
BTE	**76.43**	**0.521**

From Table 6, using STS-Gold dataset (D1), for positive class it can be seen that ANN-GD outperforms all other individual classifiers with an accuracy of 87.82%, including MVE. Considering 13 out of 14 individual classifiers that return an accuracy of >70% and constitute the Best trained ensemble, the test set data returns an accuracy of 85.83%. Though higher than MVE, it is lower than ANN-GD. However, the BTE outperforms ANN-GD on Precision, recall and F1-scores. Decision Tree performs the poorest among all classifier with 69.98% on accuracy.

From Table 7, using OMD dataset (D2), for positive class it can be seen that BTE outperforms on all parameters with an accuracy of 87.82%. In individual classifiers, SVM-Sig with 85.96% accuracy, SVM-Poly with 84.18% precision, ANN-GD with 84.39% recall and SVM-Sig with 83.66% F1-score share the top spots. ELM-TRI performs the poorest among all classifiers with 70.27% on accuracy.

From Table 8, using HCR dataset (D3), the BTE outperforms on all parameters with an accuracy of 86.09%. In individual classifiers, LOGR-LBFGS with 84.15% accuracy, SVM-Sig with 84.39% precision, SVM-Poly with 83.33% recall and SVM-Lin with 82.81% F1-score share the top spots. Again, ELM-HL performs the poorest among all classifiers with 76.04 on accuracy.

From Table 9, using SemEval 2017, Task4A dataset (D4), it can be seen that ANN-GD accuracy is the highest at 71.98% followed very closely by MVE at 71.35%. However, MVE scores over all other classifiers with values of 67.82% and 69.49% on *AvgRec* and $F{1^{PN}}$ respectively. Also, it is seen that only 2 out of 14 individual classifiers return an accuracy of ≥70% which are considered in BTE on which the test set data is run. It is observed that the BTE model outperforms all individual classifiers as well as the majority voting ensemble. The lowest performance across all metrics is achieved by ELM-T classifier.

On SemEval 2017, Task4B dataset (D5), it can be seen from Table 10 that all the individual classifiers return an accuracy of >70% and hence MVE equals BTE. The ensemble model outperforms all individual classifiers. The proposed method achieved 92.83% accuracy and average F1 of 94.74%. Among all the individual classifiers, the ANN-GD classifier achieved the highest *Acc* of 89.47% and an average F1-score of 89.75%. The lowest performance across all metrics is demonstrated by the DT classifier.

On SemEval 2017, Task4C dataset (D6), it can be seen from Table 11 that 7 out of 14 individual classifiers return an accuracy of >70% which are considered in BTE on which the test data is run. It is observed that the BTE model outperforms all individual classifiers as well as the majority voting ensemble. The proposed method achieved the highest performance in terms of *Acc* and $MA{E^M}$ (lower is better) with values of 76.43% and 0.521 respectively. Among all the individual classifiers, the ANN-GD classifier achieved the highest *Acc* and $MA{E^M}\;$with a score of 74.34% and 0.687 respectively. This is lower than the majority voting ensemble with scores of 74.88% and 0.565 respectively. The lowest performance is achieved by SVM-Lin classifier with a $MA{E^M}$ score of 0.829.

Overall, the CFS augmented SRML model with best trained ensemble strategy outperforms individual classifiers on all the datasets in Intra-model performance comparison. Among individual classifiers, ANN-GD emerges as the model of choice which along with Ensemble model can be explored for future design of a robust expert system for sentiment analysis tasks.

### Inter-model performance assessment

In Inter-model performance assessment, we compare the efficacy of the proposed model with existing state-of-the-art systems that have used similar datasets. On dataset D1, previous studies have used several advanced machine learning models for sentiment classification. We compared our findings with four recent research related to sentiment analysis and compared their accuracy. Table 12 compares the existing studies with our strategy for sentiment analysis. It is seen that the word embeddings like Sentiment-Specific Word Embedding model (SSWE), and Transformer-encoder models like BERT, RoBERTa, BERTweet return the best accuracy as seen from the results published (Barreto et al., 2021). The accuracy of the proposed model is 86.23%, though just behind the above results but it outperforms the results of the study using GloVe-DCNN (Jianqiang, Xiaolin & Xuejun, 2018), SVM and MNB along with T-conorm method (Zarisfi, Sadeghi & Eslami, 2020).

**Table 12:** Performance comparison of proposed system with state-of-the-art system on dataset D1 (Best results in bold).

Et al., Year	Model	Acc (%)
Jianqiang, Xiaolin & Xuejun (2018)	GloVe-DCNN	85.97
Zarisfi, Sadeghi & Eslami (2020)	SVM+T-conorm method	85.92
	MNB+T-conorm method	84.16
Barreto et al. (2021)	SVM SSWE static	88.99
	SVM+RoBERTa	89.48
	SVM+BERT	90.46
	SVM+BERTweet	**93.56**
Proposed	CFS augmented Best Trained Ensemble	86.23

Table 13 compares performance of the proposed model with existing systems when compared on the D2 dataset. It is observed that the proposed CFS augmented BTE model outperforms all the models compared with an accuracy of 86.82%. The top systems for this task employed QSR, BERT, BERTweet, RoBERTa and ensemble methods.

**Table 13:** Performance comparison of proposed system with state-of-the-art system on dataset D2 (Best results in bold).

Et al., Year	Model	Acc (%)
Zhang et al. (2019)	QSR-NB	65
	QSR-SVM	66
	QSR-RF	64.5
Barreto et al. (2021)	SVM+RoBERTa-static	85.10
	SVM+BERT	85.62
	SVM+BERTweet	87.36
Zarisfi, Sadeghi & Eslami (2020)	SVM+T-conorm method	87.75
	MNB+T-conorm method	84.11
Troussas, Krouska & Virvou (2016)	Ensemble of 4 base classifiers (stacking)	87.57
Proposed	CFS augmented Best Trained Ensemble	**87.82**

Table 14 compares performance of the proposed model with existing systems when compared on D3 dataset. It is observed that the proposed CFS augmented BTE model with an accuracy of 86.09% outperforms all the models compared. The top systems for this task employed NB, SVM classifiers with TF-IDF, BERT, BERTweet, RoBERTa and ensemble methods.

**Table 14:** Performance comparison of proposed system with state-of-the-art system on dataset D3 (Best results in bold).

Et al., Year	Model	Acc (%)
Bibi et al. (2020)	SVM TF-IDF	72
	NB TF-IDF	72
Barreto et al. (2021)	SVM TF-IDF	80.24
	SVM+RoBERTa	76.67
	SVM+BERT	78.61
	SVM+BERTweet	79.82
Troussas, Krouska & Virvou (2016)	Ensemble of 4 base classifiers (stacking)	85.10
Proposed	CFS augmented Best Trained Ensemble	**86.09**

Table 15 compares the proposed system with other state-of-the-art models on dataset D4 *i.e*., SemEval-2017 Task 4A. This dataset is designed for the message polarity classification task and contains three classes namely positive, negative and neutral. The top three systems for this task employed CNN, LSTM and Neural networks. The proposed CFS augmented BTE system outperforms the other models with 74.87%, 71.28% and 72.71% values on *accuracy, AvgRec* and $F{1^{PN}}$ respectively.

**Table 15:** Performance comparison of proposed system with state-of-the-art system on dataset D4 (Best results in bold).

Et al., Year, System	Acc (%)	AveRec (%)	${F1^{PN}}$ (%)
Cliche (2017). BB_twtr	65.8	68.1	68.5
Baziotis, Pelekis & Doulkeridis (2017). DataStories	65.1	68.1	67.7
Rouvier (2017). LIA	66.1	67.6	67.4
Proposed CFS augmented BTE	**74.87**	**71.28**	**72.71**

Table 16 compares the proposed system with the state-of-the-art models on dataset D5 *i.e*., SemEval-2017 Task 4B. This dataset is designed for two-point scale classification of the messages with given topics. The proposed CFS augmented BTE system outperforms all other compared models with values of 92.83%, 91.06% and 94.74% *accuracy, AvgRec* and *F1-score* respectively. The other top three systems for this task used CNNs and LSTMs with Attention model.

**Table 16:** Performance comparison of proposed system with state-of-the-art system on dataset D5 (Best results in bold).

Et al., Year, System	Acc (%)	AvgRec (%)	F1-score (%)
Cliche (2017). BB_twtr	89.7	88.2	89
Baziotis, Pelekis & Doulkeridis (2017). DataStories	86.9	85.6	86.1
Kolovou et al. (2017). Tweester	86.3	85.4	85.6
Proposed CFS augmented BTE	**92.83**	**91.06**	**94.74**

Table 17 compares the proposed system with the state-of-the-art models on dataset D6 *i.e*., SemEval-2017 Task 4C dataset. This dataset is designed for topic-based classification task with 5-point scale. The proposed CFS augmented BTE system returns an $MA{E^M}$ (lower is better) of 0.521 behind a value of 0.481 (Cliche, 2017). However, it outperforms four other state-of-the art systems compared. For this, two systems employed deep learning algorithms, TwiSe system used LR classifier and the other remaining used ensemble weighted majority vote classifier.

**Table 17:** Performance comparison of proposed system with state-of-the-art system on dataset D6 (Best results in bold).

Et al., Year, System	${\rm MAE^M}$
Rozental & Fleischer (2017). Amobee-C-137	0.599
Kolovou et al. (2017). Tweester	0.623
Balikas (2017). TwiSe	0.640
Baziotis, Pelekis & Doulkeridis (2017). DataStories	0.555
Cliche (2017). BB_twtr	**0.481**
Proposed CFS augmented BTE	0.521

## Conclusions

Considering the significance of sentiment analysis for organizations to understand consumer expectations, buying behavior and product innovations to influence decision-making, in this work a novel approach to exploit semantic features and significant features using the classifier ensemble approach was proposed. The use of classifier ensembles for Twitter sentiment analysis has been underexplored in literature. We have demonstrated that classifier ensemble formed by diversified algorithms, especially if these come from different paradigms and then choosing only the best performers out of them can provide state-of-the-art results in this domain. We also proposed a model that employs the efficacy of word2vec based continuous bag-of-words and *n*-gram feature extraction in conjunction with SentiWordNet for the representation of tweets. A novel statistical-based Cascade Feature selection approach provides optimal features that retains the overall performance of the classifiers even with reduced features. Results from the experimental study on all the six datasets, STS-Gold, OMD, HCR and the SemEval2017 Task 4A, 4B and 4C demonstrate that CFS can support in attaining a higher classification accuracy with up to 50% lesser features that augers well for large sized datasets. In Intra-model performance assessment, the ANN-GD classifier performs better compared to all other individual classifiers. However, the best trained ensemble (BTE) strategy outperforms individual classifiers on all metrics. In Inter-model performance assessment with existing state-of-the-art systems, the proposed model yielded a higher predictive accuracy on all datasets. The system outperforms compared existing classifiers like NB and SVM with TF-IDF, GloVe, T-conorm, QSR, BERT, BERTweet, RoBERTa and ensemble methods. In future works, attempts will be made one to improve the performance of SRML on D1 against transformer-based models and secondly to augment SRML with static word embeddings on many latest, large and complex datasets. Current research provides critical insights into building a generic expert system for sentiment analysis extendable to all social media platforms that can be beneficial across industries.

## Supplemental Information

10.7717/peerj-cs.1100/supp-1 Supplemental Information 1 Code for Twitter data.

10.7717/peerj-cs.1100/supp-2 Supplemental Information 2 Code for classification.

10.7717/peerj-cs.1100/supp-3 Supplemental Information 3 Code for analysis.

## Additional Information and Declarations

### Data Availability

The following information was supplied regarding data availability: The data is available at https://github.com/pmbaumgartner/text-feat-lib (D1, D2, D3), https://alt.qcri.org/semeval2017/task4/index.php?id=data-and-tools (D4, D5, D6). The codes are available in the Supplemental Files.
